# Cardiac Rehabilitation and Mortality Risk Reduction in Peripheral Artery Disease at 6-Month Outcome

**DOI:** 10.3390/diagnostics12061500

**Published:** 2022-06-20

**Authors:** Razvan Anghel, Cristina Andreea Adam, Ovidiu Mitu, Dragos Traian Marius Marcu, Viviana Onofrei, Mihai Roca, Alexandru Dan Costache, Radu Stefan Miftode, Grigore Tinica, Florin Mitu

**Affiliations:** 1 Clinical Rehabilitation Hospital, Cardiovascular Rehabilitation Clinic, Pantelimon Halipa Street nr 14, 700661 Iaşi, Romania; razvan0312@gmail.com (R.A.); adam.cristina93@gmail.com (C.A.A.); roca2m@yahoo.com (M.R.); adcostache@yahoo.com (A.D.C.); mitu.florin@yahoo.com (F.M.); 2 Department of Internal Medicine, University of Medicine and Pharmacy “Grigore T. Popa”, University Street nr 16, 700115 Iaşi, Romania; dragos.marcu11@yahoo.com (D.T.M.M.); onofreiviviana@gmail.com (V.O.); radu.miftode@yahoo.com (R.S.M.); 3 Sf. Spiridon” Clinical Emergency Hospital, Independence Boulevard nr 1, 700111 Iaşi, Romania; 4 Department of Cardiovascular Surgery, University of Medicine and Pharmacy “Grigore T. Popa”, University Street nr 16, 700115 Iaşi, Romania; grigoretinica@yahoo.com; 5 Institute of Cardiovascular Diseases ”Prof. Dr. George I.M. Georgescu”, 700503 Iași, Romania

**Keywords:** cardiac rehabilitation, peripheral artery disease, functional status, exercise, prognostic index, mortality risk

## Abstract

The management of patients with peripheral artery disease (PAD) is integrative and multidisciplinary, in which cardiac rehabilitation (CR) plays a prognostic role in terms of functional status, quality of life, and long-term impact on morbidity and mortality. We conducted a prospective cohort study on 97 patients with PAD admitted to a single tertiary referral center. Based on a prognostic index developed to stratify long-term mortality risk in PAD patients, we divided the cohort into two groups: low and low-intermediate risk group (45 cases) and high-intermediate and high risk group (52 cases). We analyzed demographics, clinical parameters, and paraclinical parameters in the two groups, as well as factors associated with cardiological reassessment prior to the established deadline of 6 months. Obesity (*p* = 0.048), renal dysfunction (*p* < 0.001), dyslipidemia (*p* < 0.001), tobacco use (*p* = 0.048), and diabetes mellitus (*p* < 0.001) are comorbidities with long-term prognostic value. Low-density lipoprotein cholesterol (*p* = 0.002), triglycerides (*p* = 0.032), fasting glucose (*p* = 0.011), peak oxygen uptake (*p* = 0.005), pain-free walking distance (*p* = 0.011), maximum walking time (*p* < 0.001), and maximum walking distance (*p* = 0.002) influence the outcome of PAD patients by being factors associated with clinical improvement at the 6-month follow-up. PAD patients benefit from enrollment in CR programs, improvement of clinical signs, lipid and carbohydrate profile, and weight loss and maintenance of blood pressure profile within normal limits, as well as increased exercise capacity being therapeutic targets.

## 1. Introduction

Atherosclerosis is the basis of the cardiovascular continuum, which includes a variety of cardiovascular diseases (CVD) such as myocardial infarction, aortic aneurysm, peripheral vascular disease or stroke [1]. Peripheral artery disease (PAD) is the result a multifactorial and chronic condition affecting the inferior vascular axis of the lower limbs with increasing prevalence and marked economic and social impact despite the medical progress made in recent decades. PAD patients are associated with multiple comorbidities, which makes it necessary to approach it with a multidisciplinary management in order to prevent complications, improve functional status, and increase quality of life [2].

Cardiac rehabilitation (CR) is an essential cornerstone in the management of these cases, along with weight management, a healthy diet, smoking cessation, and control of cardiovascular risk factors. Adherence is one of the most important elements of any CR program, the onus being on both the physician and the patient to understand the benefits on prognosis. Physical training determines morphological, hemodynamic, and metabolic changes. Improving weight, lipid, and carbohydrate profiles leads to a decrease in the degree of disability and implicitly to the growth of physical independence that improves quality of life [3,4]. Current best practice guidelines [5] recommend enrolling patients with PAD in extended CR programs to increase pain-free walking distance, walking time, and walking distance. Many programs are based on walking exercise therapy, but in selected cases alternative exercise modes such as cycling, strength training, and upper-arm ergometry may be useful.

The aim of this study was to highlight the impact of physical exercise on clinical and paraclinical parameters 6 months after the start of the CR program. At the same time, based on the 10-year mortality risk assessed using an index score, we wanted to highlight the positive, beneficial role of the multidisciplinary approach in improving the prognosis of these patients.

## 2. Materials and Methods

### 2.1. Study Design

**Figure 1:**
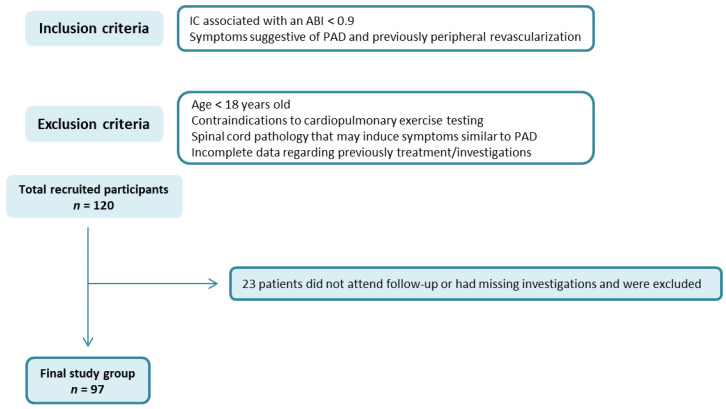
Flow chart of the studied group (IC: intermittent claudication; ABI: ankle-brachial index; PAD: peripheral artery disease).

We conducted a prospective cohort study on 120 patients admitted to a single tertiary referral center in Northeast Romania, previously diagnosed with PAD. The diagnosis of PAD was based on the presence of intermittent claudication (IC) associated with an abnormal ankle-brachial index (ABI) less than 0.9 or in the presence of symptoms suggestive of PAD and, previously, peripheral revascularization. The exclusion criteria were patients under the age of 18 years old, those with contraindications to cardiopulmonary exercise testing, those who did not attend regular check-ups, patients with associated spinal cord pathology that may induce symptoms similar to PAD, or those with incomplete data regarding treatment or investigations of interest. Of the 120 patients initially enrolled, 23 were excluded due to follow-up drop-out or due to missing investigations (Figure 1).

Patients underwent an extensive evaluation prior to enrolment in the study, which included biological samples, transthoracic echocardiographic assessment, cardiopulmonary exercise testing (CPET), and ambulatory blood pressure monitoring for 24 h. The laboratory tests performed at enrollment included: total cholesterol (mg/dL), low-density lipoprotein cholesterol (mg/dL), high-density lipoprotein cholesterol (mg/dL), triglycerides (mg/dL), blood glucose (mg/dL), glycated hemoglobin (HbA1c, %), C-reactive protein (mg/dL), and fasting glucose, using standard determination methods.

Prior to CPET, all patients were echocardiographically evaluated according to the protocols of the European Association of Cardiovascular Imaging (EACVI) to identify potential contraindications or assess cardiac function. All patients enrolled in the study underwent CPET on the cycloergometer. The main functional parameters monitored were oxygen uptake with maximal aerobic capacity (absolute value; VO_2_ max (mL per min); peak value of the respiratory exchange ratio (RER), defined as the ratio between VCO_2_ and VO_2_; maximal heart rate (HR (bpm)); and maximal systolic and diastolic blood pressure. Blood pressure was measured every 2 min during the test using the listening method, and the electrocardiogram in 12 leads was recorded until the test was stopped. Subjective assessment of fatigue that was directly proportional to the intensity of the exercises was made using the Borg scale (intensity rated from 6 to 20). CPET testing was stopped at the patients’ indication, at the onset of symptoms or muscle fatigue, in case of increased blood pressure values (systolic BP over 220 mmHg or diastolic over 120 mmHg), or when ischemic changes were observed on the electrocardiogram.

For the 97 PAD patients eligible for this study, demographics, personal medical history, tobacco, and alcohol consumption habits and chronic medication were obtained from the observation charts.

**Figure 2:**
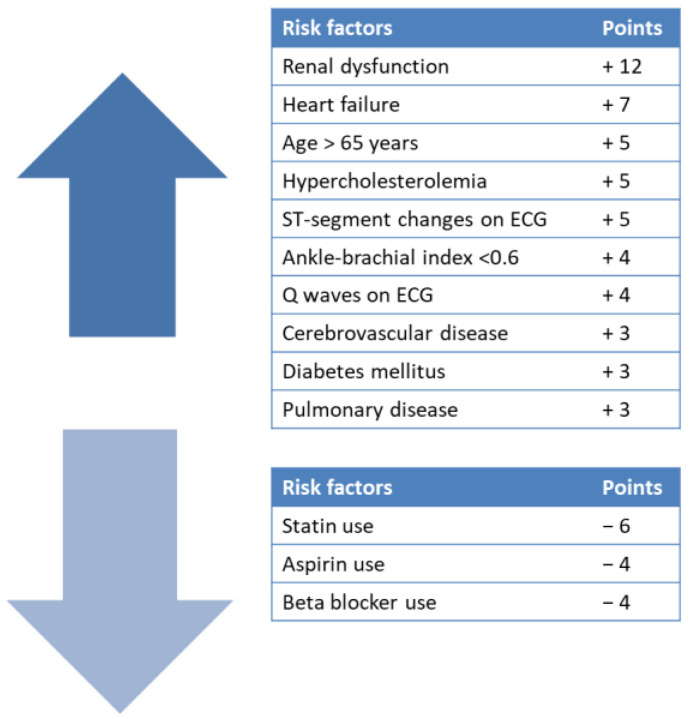
Risk index for 10-year mortality rates in patients with PAD (adapted after [11]).

We used the current guidelines to evaluate and define comorbidities, such as arterial hypertension [6], heart failure [7], chronic kidney disease [8,9], and diabetes [10]. The laboratory testing consisted of lipid profile, serum glucose, C-reactive protein (CRP), and glycated hemoglobin (HbA1C), and the results were presented according to the International System of Units. Body mass index (BMI) was calculated as the ratio of weight (kg) and height (m^2^). All patients underwent a cardiopulmonary exercise test to determine functional capacity and to assess recommended parameters for aerobic physical training. At the 6-month follow-up, biological samples, cardiopulmonary exercise testing, and blood pressure monitoring were repeated. During the CR program, all patients received dietary advice, psychotherapy, and drug therapy (statins for dyslipidemia and hypoglycemic treatment in diabetic patients). Based on the validated prognostic index developed to stratify long-term mortality risk in PAD patients [11], we calculated the scores of all patients at admission, based on which we then made the distribution in the two groups. The risk index included various parameters with a negative prognostic value—renal dysfunction (+12), heart failure (+7), ST-segment changes (+5), age over 65 years (+5), hypercholesterolemia (+5), ankle-brachial index less than 0. 60 (+4), Q waves (+4), diabetes (+3), cerebrovascular disease (+3), and lung disease (+3)—or a positive one: use of statins (−6), aspirin (−4), or β-blockers (−4). Based on the score, patients were divided into several risk categories with corresponding mortality rates: low (<0 points, 22.1%), low-intermediate (0–5 points, 32.2%), high-intermediate (6–9 points, 45.8%), and high (above 9 points, 70.4%) risk categories (Figure 2). All patients enrolled in the study were on aspirin and statin, while beta-blockers were administered to 92 patients. All patients enrolled in the study were on vasodilatory medication (pentoxifylline or cilostazol).

The exercise training program involves a protocol of 3 sessions of treadmill walking/week for 12 weeks (total of 36 sessions), with a session duration between 35 and 50 min. The speed of travel and the slope of the treadmill are initially adjusted to produce intermittent claudication 3–5 min after the onset of exercise. Patient walks until claudication is moderate to severe (intensity 4/5) and thereafter waits at rest or sitting for resolution of symptoms. When patients are able to walk 8 min or more below the claudication threshold (4/5), the difficulty level of the exercise is adjusted by increasing the slope or speed of the treadmill. Initially, the total duration of the exercise (walking and rest) is 35 min, to be increased by 5 min at each session to a total duration of 50 min.

### 2.2. Statistical Analysis

Data were reported as the mean ± SD and as a number (frequency or percentages). The Kolmogorov–Smirnov test was used to assess the normal distribution of the data. Continuous variables were compared using the *t*-test (parametric analysis). Categorical variables were compared using Fisher’s Exact test. A *p*-value of ≤ 0.05 was considered statistically significant. Receiver operating characteristic analyses were performed to calculate area under the curve for clinical parameters. The descriptive analysis was performed using SPSS statistics software (version 20 for Windows; SPSS Inc., Chicago, IL, USA).

### 2.3. Ethics

The study was approved by the Ethics Committee of the University of Medicine and Pharmacy “Grigore T. Popa” Iași and of the Clinical Rehabilitation Hospital Iași, and was conducted according to the Helsinki Declaration. All patients signed an informed consent statement, which mentioned that the results would be used for research purposes.

## 3. Results

In our study, we enrolled 97 patients with PAD and evaluated them both on admission and after 6 months of multidisciplinary management (physical training, dietary advice, psychotherapy, and control of cardiovascular risk factors). Patients were divided into two groups based on their score on the risk index for 10-year mortality rates as follows: the low and low-intermediate risk patient group (45 patients with a score of maximum 5 points) and the intermediate-high risk patient group (52 patients with a score above 6 points). The demographics and resting hemodynamics are presented in Table 1. We enrolled in the study patients with a mean age of 69.66 ± 8.60 years old, mainly men (67%). The analysis of demographic data included anthropometric parameters, the average BMI at enrolment being 27.74 ± 3.65 kg/m^2^, which increased compared to the 6-month average in both groups (25.06 ± 2.93 kg/m^2^ vs. 23.32 ± 2.96 kg/m^2^, *p* = 0.034). At the 6-month follow-up, the percentage of sedentary patients decreased compared to enrolment, with a higher percentage in the high-intermediate and high risk group (48.8% vs. 55.76%). Undertaking a personalized exercise program, changing lifestyle and adopting a healthy diet led to improvements in blood pressure (mean value for the systolic component of 129.75 ± 21.27 mmHg vs. 139.06 ± 19.24 mmHg, *p* = 0.054 and for the diastolic component of 75.98 ± 14.47 mmHg vs. 78.38 ± 14.46 mmHg, *p* = 0.418) and heart rate (68.83 ± 10.98 bpm vs. 64.49 ± 9.13 bpm, *p* = 0.054). The integrative management approach also led to the correction of cardiovascular risk factors represented by smoking cessation (20.0% vs. 38.5%, *p* = 0.048) or alcohol consumption (4.4% vs. 13.5%, *p* = 0.130). Improvement in anthropometric parameters has also led to a reduction in the percentage of obese patients with a total of 73.3% patients in the low and low-intermediate risk group, respectively 53.8% in the high-intermediate and high risk group.

Physical training and correction of cardiovascular risk factors also led to clinical improvement in terms of paresthesia (31.1% vs. 53.8%, *p* = 0.015), feeling of cold feet (37.8% vs. 63.5%, *p* = 0.011), pale skin (24.4% vs. 46.2%, *p* = 0.026), and cold skin (22.2% vs. 44.2%, *p* = 0.022).

The prognostic index included comorbidities and paraclinical parameters alike, which are presented in Table 2. Patients in the high-intermediate and high risk group were more frequently associated with renal dysfunction (8.9% vs. 94.2%, *p* < 0.001), dyslipidemia (40.0% vs. 75.0%, *p* < 0.001), an ABI value below 0.6 (37.8% vs. 44.2%, *p* = 0.525), cerebrovascular disease (31.1% vs. 44.2%, *p* = 0.188), diabetes mellitus (28.9% vs. 82.7%, *p* < 0.001), patients over 65 years old (44.4% vs. 53.8%, *p* = 0.361), and pulmonary diseases (6.7% vs. 21.2%, *p* = 0.043). Heart failure (80.0% vs. 65.4%, *p* = 0.111) and electrocardiographic changes were identified more frequently in patients in the low and low-intermediate risk group (ST-segment changes, *p* = 0.039 and Q-waves, *p* =0.306). The management of patients with PAD enrolled in the study included a multidisciplinary approach, adapted to the particularities and comorbidities of each patient. Thus, statins and aspirin were administered to all patients. Beta-blocker therapy was administered to 92 patients (94.84%, 42 in the first group and 50 in the second group). Renin-angiotensin system blockers were administered to 84.44% of the low and low-intermediate risk group and 96.15% of the high-intermediate and high risk group. Patients with diabetes mellitus were treated with oral antidiabetics (100% in the first group and 88.47% in the second group) or insulin (none in the first group and 11.53% in the second group) (Table 3).

Table 4 and Table 5 show the biological patient characteristics and the exercise stress test parameters. All patients benefited from a multidisciplinary approach, with weight loss, healthy diet, and adherence to drug treatment being factors associated with improved lipid and carbohydrate profiles. Statistically significant results were obtained for low-density lipoprotein (LDL) cholesterol (101.96 ± 41.73 mg/dL vs. 130.97 ± 48.90 mg/dL, *p* = 0.002) and triglycerides (132.19 ± 63.12 mg/dL vs. 180.29 ± 126.53 mg/dL, *p* = 0.032), as well as fasting glucose (126.09 ± 31.59 mg/dL vs. 153.30 ± 63.44 mg/dL, *p* = 0.011) or HbA1C (7.08 ± 1.85 g% vs. 7.92 ± 1.94 g%, *p* = 0.03).

Cardiopulmonary exercise testing performed 6 months after the start of physical training showed an improvement in functional parameters. Peak oxygen uptake (VO_2peak_) (15.96 ± 6.07 vs. 12.88 ± 4.52, *p* = 0.005), peak systolic blood pressure (152.28 ± 19.08 mmHg vs. 161.83 ± 19.83 mmHg, *p* = 0.018), and perception of effort on the Borg scale (13.64 ± 1.03 vs. 13.02 ± 1.16, *p* = 0.034) are statistically significant parameters in our study. Mean values for ABI (0.87 ± 0.25 vs. 0.76 ± 0.26, *p* = 0.034) and left ventricular ejection fraction (LVEF, %) (51.47 ± 11.25 vs. 47.35 ± 9.71, *p* = 0.056) were improved following physical training and secondary preventive measures. The clinical improvement of the patients occurred secondary to the improvement of the functional status assessed through pain-free walking distance (*p* = 0.011), maximum walking time (*p* < 0.001), and maximum walking distance (*p* = 0.002).

**Figure 3:**
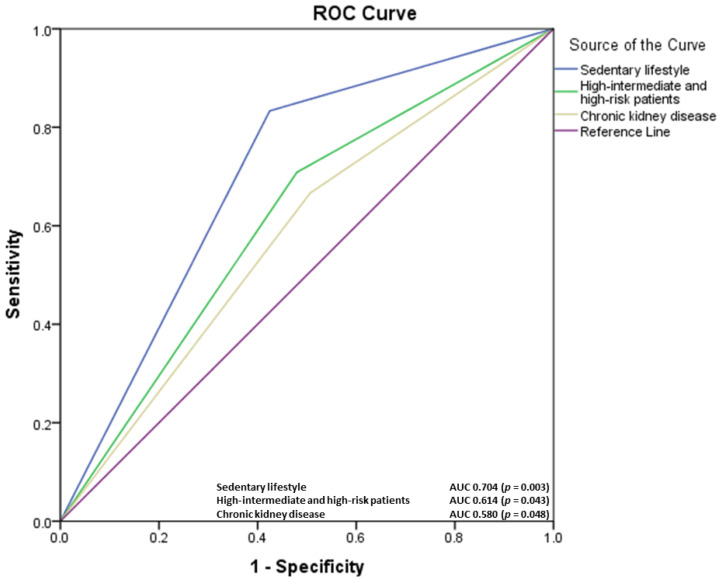
The receiver operating characteristic curve of factors associated with specialist reassessment before the 6-month assessment period.

A total of 15.6% of the patients of the first group and 32.7% of the patients of the second group were cardiologically re-evaluated in the first 6 months after starting physical training and experiencing worsening of symptoms (especially dyspnea), fatigue, chest pain, or blood pressure oscillations. Among the risk factors included in the prognostic risk, a positive correlation with cardiological reassessment (*p* < 0.05) was identified for sedentary lifestyle, chronic kidney disease, heart failure, age above 65 years, ST-segment changes, diabetes mellitus, ABI values less than 0.6 and high-intermediate and high risk patients. The receiver operating characteristic (ROC) analysis was performed for the parameters mentioned above, with the most significant predictors shown in the figure. The ROC curve of factors associated with specialist reassessment before the 6-month assessment period identified positive predictive values for high-intermediate and high risk group patients (area under the curve <AUC> = 0.614, *p* = 0.043), sedentary lifestyle (AUC = 0.704, *p* = 0.003), diabetes mellitus (AUC = 0.559), obesity (AUC = 0.553), heart failure (AUC = 0.574), and chronic kidney disease (AUC = 0.580, *p* = 0.048) (Figure 3).

## 4. Discussions

Atherosclerosis is a multifactorial process, in which genetic, environmental, and classical cardiovascular risk factors underlie complex pathophysiological processes. Progression of atherosclerotic lesions occurs over time and the removal of modifiable risk factors is essential for a favorable prognosis [12]. Cardiac rehabilitation plays an essential role in the management of PAD’s essential role in improving prognosis by decreasing morbidity and mortality [13]. Cardiac rehabilitation during the COVID-19 pandemic was a challenge for both medical staff and patients in light of social distance or work-related restrictions [3,14].

Using a validated prognostic index developed to stratify long-term mortality risk in PAD patients, we evaluated the clinical and paraclinical factors associated with improved prognosis secondary to enrollment in a CR program. Additionally, we wanted to highlight the importance of multidisciplinary management in PAD, the benefit being multiplicative by correcting cardiovascular risk factors.

We enrolled in our study predominantly male patients, with an average age of 69.66 ± 8.60 years. A total of 44.4% of the low and low-intermediate risk group and 53.8% of those with high-intermediate and high risk (*p* = 0.361) were over 65 years of age. The prevalence of PAD increases with age, ranging from 1% in patients aged 40 to 49 to 15% in patients over 70 [15]. Men have a higher risk of developing atherosclerosis disease and, over time, a higher risk of showing specific clinical signs of PAD, CAD, or stroke [16,17].

Patients in the high-intermediate and high risk group had more comorbidities and cardiovascular risk factors than those whose mortality risk was assessed as being low or low-intermediate. In our study, we concluded that renal dysfunction (*p* < 0.001), dyslipidemia (*p* < 0.001), ST-segment change (*p* = 0.039), diabetes mellitus (28.9% vs. 82.7%, *p* < 0.001), and pulmonary diseases (6.7% vs. 21.2%, *p* = 0.043) have a negative prognostic role associated with a significant mortality rate 10 years after diagnosis. Patients with diabetes mellitus, high serum levels of triglycerides and microvascular disease, or smokers present more frequently with PAD rather than CAD or stroke [15,17,18]. Recently, Lu et al. [19] demonstrated that increases in systolic blood pressure values above 140 mmHg are associated with a 2.6-fold increased risk of developing PAD, similarly to those with values between 120 and 139 mmHg that have an associated risk of 1.6. In the case of the diastolic component, the investigators observed that values above 90 mmHg have a negative prognostic role.

Diabetes mellitus is one of the most important risk factors for the onset and progression of PAD, with microvascular or macrovascular complications having a negative impact on morbidity and mortality through the associated functional decline and decreased quality of life [20,21]. The risk of chronic limb-threatening ischemia (CTLI) or amputations depends on the HbA1c value [22]. In our study, we demonstrated that patients with high-intermediate and high risk had higher serum HbA1c values (7.08 ± 1.85 % vs. 7.92 ± 1.94 %, *p* = 0.003), which are associated with a negative prognosis both in the short- and long-term acceleration of the atherosclerotic process. Geiss et al. demonstrated that diabetic patients with inadequate glycemic control (HbA1c ≥ 7%) compared to those with no diagnosis of diabetes have a relative risk of developing CTLI (10.3) that negatively influences long-term prognosis [23].

Along with diabetes, smoking is another risk factor with a negative prognostic impact in PAD, being associated with a 2–3-fold relative risk increase. In our study, smoking patients received psychotherapy and counselling on the importance of correcting this cardiovascular risk factor associated with an increased rate of long-term complications and thus an increased risk of death, with the 6-month evaluation showing a decrease in their percentage from 20.0% in the low and low-intermediate risk group to 38.5% in the high-intermediate and high risk group (*p* = 0.030). The risk of smoking patients to develop PAD and not CAD or stroke was demonstrated in a recent Mendelian randomization analysis by Levin et al. [24]. Dratva et al. highlighted the negative impact of smoking in young people by increasing carotid intima-media thickness [25].

Between PAD and cerebrovascular disease there is an interdependent relationship considering the generalized atherosclerotic impairment. The association of other cardiovascular risk factors, such as high BP, diabetes mellitus, smoking, dyslipidemia, atrial fibrillation, or heart failure increase the risk of developing an acute event [26]. Similar results to our study were also obtained by Farag et al., who prospectively analyzed a cohort of 100 patients and concluded that the prevalence of PAD among this type of patients is high (42.0% of the cases), with asymptomatic patients (but with associated atherosclerotic lesions or an ABI value below 0.9) being associated with a higher risk of recurrent ischemic events (asymptomatic PAD group 19% vs. non PAD group 5% vs. symptomatic PAD group 4.8%) or death than symptomatic patients or those without PAD [27]. ABI value influences patients’ performance in physical training. Thus, while no limitations were observed in the case of light physical training, in the case of patients who practiced intense physical training, a limitation in the improvement of functional parameters was observed [28]. In a similar study, Sakamoto et al. studied the impact of a 12-week supervised exercise program on cardiovascular mortality and morbidity and concluded that risk of death secondary to an acute event was higher for patients who did not complete the program (90.2% vs. 85.8% at 3 years; 85.7% vs. 63.7% at 5 years; and 45.7% vs. 38.0% at 10 years, respectively, *p* = 0.401). Using multivariate regression, the investigators showed that age over 71 years, diabetes mellitus, maximum walking distance, history of coronary revascularization, and completion of a training program are independent predictors of cardiovascular fatal events.

In our study, the beneficial role of physical training in decreasing functional decline, increasing the independence of PAD patients and thus improving quality of life, can also be seen in the improvement of pain-free walking distance (*p* = 0.006), walking time (*p* < 0.001), and maximum walking distance (*p* = 0.002). Siercke et al. enrolled 118 PAD patients with IC and obtained statistically significant results 6 months after the start of the CR program for maximum walking distance (odds ratio 1.37, 95% confidence interval 1.10–1.70, *p* = 0.005), as well as for the percentage of patients who started to embrace an active lifestyle (29% vs. 51%, *p* = 0.002). An improvement in walking distance without CI was also observed (115 m vs. 133.5 m, *p* = 0.060), with a value at the limit of statistical significance [29].

On the other hand, heart failure (80.0% vs. 65.4%, *p* = 0.111) and electrocardiographic changes were identified more frequently in patients in the low and low-intermediate risk group (ST-segment changes, *p* = 0.039 and Q-waves, *p* = 0.306). PAD patients are at risk of developing heart failure, with ABI playing an important role in risk stratification, as it has been shown that the presence of a pathological value is associated with a 40% risk of progression to heart failure after correction for certain cofactors such as diabetes mellitus, smoking, CAD, or cerebrovascular disease [30,31]. Prasada et al. demonstrated in a recent study that the prognostic impact is also associated with the type of heart failure (reduced ejection fraction vs. preserved ejection fraction), as well as the associated treatment, as it is known that SGLT2 inhibitors decrease the risk of heart failure independently of the presence or absence of diabetes mellitus. ABI values correlate better with heart failure with reduced ejection fraction [31,32].

Patients with PAD are associated with a high risk of mortality or morbidity through acute vascular events or multiple associated comorbidities, polymedication, lack of adherence to medical treatment, aspects that over time lead to worsening functional decline, and decreased quality of life. Fowkes et al. demonstrated that ABI correlates significantly statistically with mortality [33]. In our study, physical training led to ABI improvement in both groups statistically analyzed (0.87 ± 0.25 vs. 0.76 ± 0.26, *p* = 0.034), clinically translated by the improvement of clinical signs, such as paresthesia (*p* = 0.015), feeling of cold feet (*p* = 0.011), pale skin (*p* = 0.026), and cold skin (*p* = 0.022). The prognostic value of ABI has been certified in multiple studies. Fowkes et al. demonstrated in a meta-analysis based on 16 patient cohorts (48,294 subjects) that the 10-year mortality rate depended on the ABI value. Thus, in patients with ABI values below 0.90, the mortality rate was 18.7% in men and 12.6% in women (compared to 4.4% and 4.1%, respectively, in cases with normal values). Additionally, in each Framingham risk score category, a low ABI value doubled the 10-year total mortality, cardiovascular mortality, and major coronary event rates compared to the overall rate [22,34]. Functional status also influences quality of life, with the presence of an ABI value less than 0.9 or within 0.9–1.0 being associated with a relatively double risk of impaired physical function [35].

The patients enrolled in the study presented in this paper benefited from multidisciplinary management, including physical training, counseling on adopting a healthy lifestyle, smoking cessation, and weight management, as well as drug treatments targeting associated comorbidities and identified cardiovascular risk factors. The prognostic index used to stratify long-term mortality risk in PAD patients included statin, aspirin, and beta-blockers as parameters with favorable prognostic value. All patients enrolled in the study received lipid-lowering medication, which led to a decrease in mean serum values of lipid profile parameters (LDL-cholesterol, *p* = 0.002 and triglycerides, *p* = 0.032). Similar results were observed for the carbohydrate profile where decreases in mean serum values were targeted for fasting glucose (*p* = 0.003) and HbA1c (*p* = 0.011). Lipid-lowering medication and drug treatment of diabetes mellitus improve the prognosis of patients with PAD, leading to decreased mortality rate or cardiac hospitalizations [36,37]. Statistical analysis revealed a more pronounced decrease in biological parameters among patients in the low and low-intermediate risk group, which should be correlated with the increased proportion of comorbidities and cardiovascular risk factors in the high-intermediate and high risk group.

Properly conducted physical training tailored to the needs and abilities of each individual patient correlates to a positive prognosis both in the short and long term. Decreased risk of acute cardiac or vascular events translates to decreased risk of morbidity and mortality and thus increased quality of life. Cardiopulmonary testing performed 6 months after the start of the CR program showed a significant increase in VO_2_ peak in patients of both groups (15.96 ± 6.07 mL/kg/min vs. 12.88 ± 4.52 mL/kg/min, *p* = 0.005). VO_2_ peak is a marker for aerobic exercise capacity and an independent predictor factor for all-cause mortality and cardiac mortality in PAD [38,39]. The physical performance of patients in the high-intermediate and high risk group was inferior, a fact explained by the higher degree of disability associated with multiple comorbidities, older age, and previous physical deconditioning. The simultaneous increase in VO_2_ peak and LVEF in the second group suggests the beneficial role of enrolling these patients in CR programs. In a similar study, Vanhees et al. demonstrated that baseline exercise performance and training characteristics are the strongest determinants of further evolution, with the use of antiarrhythmics (odds ratio 5.5, *p* < 0.001) and ST-segment depression of at least 1 mm (odds ratio 1.6, *p* < 0.001) being the eloquent risk factors associated with a high risk of complications [40]. As mentioned before, the clinical benefits of physical training were an improvement in clinical signs, ABI value, and Borg scale score (13.64 ± 1.03 vs. 13.02 ± 1.16, *p* = 0.006).

A total of 15.6% of the patients in the low and low-intermediate risk group and 32.7% of the patients in the high-intermediate and high risk group were reassessed at 6 months after starting physical training. The clinical picture of the patients in the second group, multiple comorbidities, and associated cardiovascular risk factors explain the percentage.

Our study presents several limitations due to the number of cases analyzed and variability in the assessment of the improvement of clinical signs. We excluded those records where critical information was unavailable. This was done to minimize the risk of misclassification, introducing a limited risk of selection bias. In addition, the clinical improvement must be interpreted through the perspective of a multidisciplinary approach, physical training, dietary advices, weight management, and medical treatment being contributing factors, with a multiplicative beneficial prognostic role in our study. As well, the 6-month limited time monitorization data may be modified for a longer follow-up.

## 5. Conclusions

In our study we demonstrated the beneficial role of enrolling PAD patients in CR programs, regardless of the mortality risk assessed at a distance, in the context of an unfavorable outcome. Improvement of clinical signs and lipid and carbohydrate profiles, normalization of weight and blood pressure profiles, and increased exercise capacity are prognostic therapeutic targets in this category of patients. Correction of risk factors and adherence to drug treatment improves quality of life and thus long-term prognosis. The development of an integrative algorithm to evaluate the functional and clinical benefits of CR programs as well as studying the prognostic role of biomarkers are future research directions for us.

## Figures and Tables

**Table 1:** Demographics, resting hemodynamics, and clinical parameters.

Parameter	Before CR (*n* = 97)	Low & Low-Intermediate Risk Group (*n* = 45)		High-Intermediate & High Risk Group (*n* = 52)		*p* Value
		Before CR	After CR	Before CR	After CR	
**Demographics**						
Males	65 (67.0%)		-		-	-
Age, y	69.66 ± 8.60	63.62 ± 10.32	-	70.81 ± 9.42	-	-
Height, cm	171.44 ± 8.77	170.51 ± 8.94	-	172.75 ± 8.18	-	-
Weight, kg	83.72 ± 15.49	74.08 ± 13.81	69.34 ± 10.98	72.14 ± 13.52	64.07 ± 12.93	0.034
Body mass index, kg/m^2^	27.74 ± 3.65	28.85 ± 4.01	25.06 ± 2.93	26.57 ± 3.43	23.32 ± 2.96	0.005
**Resting hemodynamics**						
Heart rate, bpm	72.71 ± 12.28	73.31 ± 14.03	68.83 ± 12.68	71.12 ± 10.53	64.49 ± 9.13	0.054
Systolic BP, mmHg	140.79 ± 21.56	144.63 ± 20.88	129.75 ± 21.27	141.34 ±19.98	139.06 ± 19.24	0.026
Diastolic BP, mmHg	80.15 ± 14.97	81.04 ± 14.68	75.98 ± 14.47	81.81 ± 14.65	78.38 ± 14.46	0.418
**Sedentary lifestyle**	69 (71.1%)	29 (64.44%)	22 (48.8%)	40 (76.92%)	29 (55.76%)	0.030
**Smoking**						
Active/in the past	63 (64.9%)	21 (46.66%)	9 (20.0%)	42 (80.76%)	20 (38.5%)	0.048
**Alcohol**	16 (16.5%)	5 (11.11%)	2 (4.4%)	11 (21.15%)	7 (13.5%)	0.130
**Obesity**	69 (71.1%)	36 (80.0%)	33 (73.3%)	33 (63.5%)	28 (53.8%)	0.048
**Clinical signs**						
Paresthesia	75 (77.3%)	35 (77.8%)	14 (31.1%)	40 (76.9%)	29 (55.8%)	0.015
Feeling of cold feet	68 (70.1%)	27 (60.0%)	17 (37.8%)	41 (78.8%)	33 (63.5%)	0.011
Pale skin	61 (62.9%)	25 (55.6%)	11 (24.4%)	36 (69.2%)	24 (46.2%)	0.026
Cold skin	47 (48.5%)	22 (48.9%)	10 (22.2%)	25 (48.1%)	23 (44.2%)	0.022
Reduced pilosity	36 (37.1%)	18 (40%)	17 (37.8%)	18 (34.61%)	15 (28.8%)	0.351
Subcutaneous atrophy	22 (22.68%)	9 (21.42%)	9 (20.0%)	13 (25%)	12 (23.1%)	0.714
Thickened nails	22 (22.7%)	10 (23.8%)	10 (22.2%)	12 (23.07%)	12 (23.1%)	0.920
Petechiae	32 (33.0%)	17 (37.8%)	8 (17.8%)	15 (28.8%)	13 (25.0%)	0.389
Arterial ulcers	5 (5.2%)	2 (4.4%)	1 (2.2%)	3 (5.8%)	4 (7.7%)	0.450
Dermatitis	14 (14.4%)	8 (17.8%)	5 (11.1%)	6 (11.5%)	7 (13.5%)	0.726

All values are expressed as mean ± standard deviation (SD) or *n* (%); y: years; bpm: beats per minute; BP: blood pressure.

**Table 2:** Risk factors included in the prognostic index.

Risk Factors	Low & Low-Intermediate Risk Group (*n* = 45)	High-Intermediate & High Risk Group (*n* = 52)	*p* Value
Renal dysfunction	4 (8.9%)	49 (94.2%)	<0.001
Heart failure	36 (80.0%)	34 (65.4%)	0.111
Age > 65 years	20 (44.4%)	28 (53.8%)	0.361
Hypercholesterolemia	18 (40.0%)	39 (75.0%)	<0.001
ST-segment changes	31 (68.9%)	25 (48.1%)	0.039
ABI <0.6	17 (37.8%)	23 (44.2%)	0.525
Q-waves	22 (48.9%)	20 (38.5%)	0.306
Cerebrovascular disease	14 (31.1%)	23 (44.2%)	0.188
Diabetes mellitus	13 (28.9%)	43 (82.7%)	<0.001
Pulmonary disease	3 (6.7%)	11 (21.2%)	0.043

All values are expressed as *n* (%).

**Table 3:** Pharmacological treatment.

Therapeutic Agents	Low & Low-Intermediate Risk Group (*n* = 45)	High-Intermediate & High Risk Group (*n* = 52)
Beta-blockers	42 (93.3%)	50 (96.15%)
Renin-angiotensin system blockers	38 (84.44%)	50 (96.15%)
Oral antidiabetics	45 (100%)	47 (88.47%)
Insulin	0 (0%)	5 (11.53%)
Statins	45 (100%)	52 (100%)
Aspirin	45 (100%)	52 (100%)

**Table 4:** Blood biochemistry and exercise stress test parameters before CR.

Parameter	Before CR (*n* = 97)	Low & Low-Intermediate Risk Group (*n* = 45)	High-Intermediate & High Risk Group (*n* = 52)
**Blood biochemistry**			
Total cholesterol, mg/dL	206.95 ± 75.90	217.72 ± 74.32	198.03 ± 78.85
LDL-cholesterol, mg/dL	135.40 ± 67.79	140.44 ± 64.44	131.05 ± 71.14
HDL-cholesterol, mg/dL	42.84 ± 16.92	43.63 ± 16.87	42.17 ± 17.11
Triglycerides, mg/dL	185.27 ± 122.13	186.57 ± 93.75	184.15 ± 143.16
CRP, mg/dL	5.33 ± 14.30	3.55 ± 4.19	6.87 ± 19.10
HbA1C, g%	7.96 ± 2.23	6.23 ± 0.87	9.92 ± 1.60
Fasting glucose, mg/dL	153.94 ± 63.54	128.55 ± 29.17	166.30 ± 77.72
**Exercise stress test**			
VO_2peak_ mL/kg/min	12.34 ± 4.74	12.06 ± 4.27	12.59 ± 5.14
Peak HR, bpm	118.61 ± 18.81	119.49 ± 18.66	116.73 ± 19.13
Peak systolic BP, mmHg	171.52 ± 27.03	174.47 ± 26.31	170.58 ± 27.91
Peak diastolic BP, mmHg	90.54 ± 13.31	89.36 ± 14.14	91.58 ± 12.61
RER	1.07 ± 0.10	1.07 ± 0.11	1.08 ± 0.10
Borg scale	15.45 ± 1.58	15.16 ± 1.48	15.02 ± 1.87
**ABI**	0.65 ± 0.21	0.74 ± 0.21	0.70 ± 0.23
**Pain free walking distance, m**	246.43 ± 185.49	220.44 ± 159.09	165.19 ± 124.88
**Walking time, min**	24.75 ± 9.15	29.50 ± 8.41	20.63 ± 7.72
**Walking distance, m**	253.27 ± 161.77	285.81 ± 153.67	225.12 ± 124.88
**Echocardiography**			
LVEF, %	46.41 ± 9.74	47.60 ± 9.45	45.13 ± 10.82

All values are expressed as mean ± standard deviation (SD). LDL-cholesterol: low-density lipoprotein cholesterol, HDL-cholesterol: high-density lipoprotein cholesterol; CRP: C reactive protein; HbA1C: glycated hemoglobin; VO_2_ peak: peak oxygen uptake; bpm: beats per minute; HR: heart rate; BP: blood pressure; RER: respiratory exchange ratio; ABI: ankle-brachial index; LVEF: left ventricle ejection fraction.

**Table 5:** Blood biochemistry and exercise stress test parameters.

Parameter	6 Months Follow-Up (*n* = 97)	Low & Low-Intermediate Risk Group (*n* = 45)	High-Intermediate & High Risk Group (*n* = 52)	*p* Value
**Blood biochemistry**	°	°	°	°
Total cholesterol, mg/dL	172.92 ± 63.12	165.96 ± 65.85	180.98 ± 59.53	0.245
LDL-cholesterol, mg/dL	121.86 ± 61.01	101.96 ± 41.73	130.97 ± 48.90	0.002
HDL-cholesterol, mg/dL	44.56 ± 17.60	45.37 ± 17.54	43.86 ± 17.79	0.675
Triglycerides, mg/dL	161.18 ± 106.26	132.19 ± 63.12	180.29 ± 126.53	0.032
CRP, mg/dL	3.42 ± 5.07	2.40 ± 2.78	4.32 ± 6.33	0.063
HbA1C, g%	7.53 ± 1.93	7.08 ± 1.85	7.92 ± 1.94	0.003
Fasting glucose, mg/dL	140.67 ± 52.73	126.09 ± 31.59	153.30 ± 63.44	0.011
**Exercise stress test**				
VO_2peak_ mL/kg/min	14.31 ± 4.74	15.96 ± 6.07	12.88 ± 4.52	0.005
Peak HR, bpm	131.66 ± 20.88	134.14 ± 18.16	128.59 ± 21.62	0.178
Peak systolic BP, mmHg	157.17 ± 19.78	152.28 ± 19.08	161.83 ± 19.83	0.018
Peak diastolic BP, mmHg	88.73 ± 13.04	87.57 ± 13.85	86.75 ± 12.36	0.415
RER	1.09 ± 0.10	1.09 ± 0.12	1.10 ± 0.11	0.695
Borg scale	13.31 ± 1.14	13.64 ± 1.03	13.02 ± 1.16	0.006
**ABI**	0.81 ± 0.25	0.87 ± 0.25	0.76 ± 0.26	0.034
**Pain free walking distance, m**	274.96 ± 175.52	323 ± 170.36	233.30 ± 170.87	0.011
**Walking time, min**	28.46 ± 10.53	33.93 ± 9.67	23.72 ± 8.88	<0.001
**Walking distance, m**	300.58 ± 187.48	361.64 ± 180.55	247.75 ± 178.61	0.002
**Echocardiography**				
LVEF, %	49.37 ± 10.43	51.47 ± 11.25	47.35 ± 9.71	0.056

All values are expressed as mean ± standard deviation (SD). LDL-cholesterol: low-density lipoprotein cholesterol, HDL-cholesterol: high-density lipoprotein cholesterol; CRP: C-reactive protein; HbA1C: glycated hemoglobin; VO_2_ peak: peak oxygen uptake; bpm: beats per minute; HR: heart rate; BP: blood pressure; RER: respiratory exchange ratio; ABI: ankle-brachial index; LVEF: left ventricle ejection fraction.
